# Imaging lung tumor motion using integrated‐mode proton radiography—A phantom study towards tumor tracking in proton radiotherapy

**DOI:** 10.1002/mp.17508

**Published:** 2024-11-12

**Authors:** Ryan Fullarton, Mikaël Simard, Lennart Volz, Allison Toltz, Savanna Chung, Christoph Schuy, Daniel G. Robertson, Gary Royle, Sam Beddar, Colin Baker, Christian Graeff, Charles‐Antoine Collins‐Fekete

**Affiliations:** ^1^ Department of Medical Physics and Biomedical Engineering University College London London UK; ^2^ Biophysics GSI Helmholtz Centre for Heavy Ion Research GmbH Darmstadt Germany; ^3^ Department of Radiotherapy Physics University College London Hospital NHS Foundation Trust London UK; ^4^ Division of Medical Physics Department of Radiation Oncology Mayo Clinic Arizona Phoenix Arizona USA; ^5^ Department of Radiation Physics The University of Texas MD Anderson Cancer Center Houston Texas USA

**Keywords:** intrafraction motion, interplay effect, proton radiography

## Abstract

**Background:**

Motion of lung tumors during radiotherapy leads to decreased accuracy of the delivered dose distribution. This is especially true for proton radiotherapy due to the finite range of the proton beam. Methods for mitigating motion rely on knowing the position of the tumor during treatment.

**Purpose:**

Proton radiography uses the treatment beam, at an energy high enough to traverse the patient, to produce a radiograph. This work shows the first results of using an integrated‐mode proton radiography system to track the position of moving objects in an experimental phantom study; demonstrating the potential of using this method for measuring tumor motion.

**Methods:**

Proton radiographs of an anthropomorphic lung phantom, with a motor‐driven tumor insert, were acquired approximately every 1 s, using tumor inserts of 10, 20, and 30 mm undergoing a known periodic motion. The proton radiography system used a monolithic scintillator block and digital cameras to capture the residual range of each pencil beam passing through the phantom. These ranges were then used to produce a water equivalent thickness map of the phantom. The centroid of the tumor insert in the radiographs was used to determine its position. This measured position was then compared to the known motion of the phantom to determine the accuracy.

**Results:**

Submillimeter accuracy on the measurement of the tumor insert was achieved when using a 30 mm tumor insert with a period of 24 s and was found to be improved for decreasing motion amplitudes with a mean absolute error (MAE) of 1.0, 0.9, and 0.7 mm for 20, 15, and 10 mm respectively. Using smaller tumor inserts reduced the accuracy with a MAE of 1.8 and 1.9 mm for a 20 and 10 mm insert respectively undergoing a periodic motion with an amplitude of 20 mm and a period of 24 s. Using a shorter period resulted in significant motion artifacts reducing the accuracy to a MAE of 2.2 mm for a 12 s period and 3.1 mm for a 6 s period for the 30 mm insert with an amplitude of 20 mm.

**Conclusions:**

This work demonstrates that the position of a lung tumor insert in a realistic anthropomorphic phantom can be measured with high accuracy using proton radiographs. Results show that the accuracy of the position measurement is the highest for slower tumor motions due to a reduction in motion artifacts. This indicates that the primary obstacle to accurate measurement is the speed of the radiograph acquisition. Although the slower tumor motions used in this study are not clinically realistic, this work demonstrates the potential for using proton radiography for measuring tumor motion with an increased scanning speed that results in a decreased acquisition time.

## 1 INTRODUCTION

Proton therapy has been shown to reduce cardiac toxicities compared to photon based radiotherapy,^1^, ^2^ in the treatment of lung cancers, which has been linked to improved outcomes.^3^, ^4^, ^5^ Radiotherapy for lung cancer requires effective management of tumor motion to achieve an accurate delivery of the planned dose distribution.^6^ This is especially true in proton therapy, where changes in water equivalent thickness (WET) of the beam path greatly alter the depth of dose deposition.^7^ Additionally, in modern pencil beam scanning systems, the interplay effect between the tumor motion and beam scanning must be considered.^8^, ^9^ Tumor motion due to breathing has been found to depend on location, volume, and clinical staging and cannot be accounted for on a general basis, therefore relying on patient specific observations.^10^, ^11^, ^12^

Current methods of managing lung tumor motion in radiotherapy include the use of 4DCT, breath‐hold or abdominal compression.^13^ A 4DCT is acquired pre‐treatment to assess motion at the planning stage. The most straightforward way to mitigate the target motion is by combining the target volumes throughout the patient motion into an internal target volume (ITV).^14^ This method assumes that the breathing motion captured during pre‐treatment is representative of the breathing motion throughout treatment and does not account for differences in tumor volume or weight loss throughout the course of treatment.^15^ Breath‐hold relies on halting or reducing the patient's breathing motion, by coaching (passive) or by using external equipment (active), with the aim of holding the tumor in the desired treatment position.^16^ Similarly, compression uses external equipment to physically restrain the patient's breathing motion. These techniques have shown effectiveness for some types of lung tumor, and their use requires careful setup to ensure dense areas of the device do not obstruct the beam.^17^, ^18^

Further to the above methods, prospective respiratory gating aims to predict the motion of the tumor during treatment and only deliver when the tumor is in the intended position. In practice, this is done with an external marker on the patient's chest, acting as a surrogate of tumor motion, which is known to not always be representative of the internal motion.^6^ Surface‐guided methods use the surface of the patient as a surrogate for internal motion. However, a low correlation has been seen between surface motion and the internal tumor motion for lung cancer.^19^, ^20^

Alternatively, the ability to dynamically track targets and alter the treatment beams accordingly has been explored and implemented in *x*‐ray radiotherapy.^21^, ^22^, ^23^ Beam tracking has also been explored in particle therapy. However, due to the need for not only lateral, but also longitudinal correction of the Bragg peak position, beam tracking is much more complicated and has not reached widespread application.^24^ Recent works have therefore relied on combining 4D optimized treatment planning, that is, generating a plan that is conformal with respect to expected target motion from the planning 4DCT, with residual tracking to account for irregular motion during treatment delivery.^25^ The required internal target motion can be extracted from *x*‐ray fluoroscopy, but the high imaging dose required to track respiratory motion poses a hurdle to continuous tracking during the treatment. Due to the poor visibility of the tumor in *x*‐ray radiography, implanted markers, such as gold fiducials^26^ are used to identify the tumor position. The use of fiducial markers not only requires patients to undergo an invasive procedure but runs the risk of drift between surrogate and actual internal motion over the course of treatment.^27^ Additionally, metal artifacts can make image guidance more difficult.^28^ Markerless tracking in *x*‐ray fluoroscopy has been explored with the use of Artificial Intelligence but further studies are required for clinical efficacy.^29^, ^30^

Proton radiography has potential as a noninvasive tool for directly monitoring tumor motion during particle radiotherapy due to its improved soft‐tissue contrast compared to *x*‐ray radiography^31^ which would not require the implant of radiological markers. Proton radiography makes use of energies higher than those typically used for treatment, to transmit the beam through the patient and use its energy loss to produce images. Previously proposed use cases are characterizing the relative stopping power (RSP) of the patient's tissues, through the acquisition of proton CT^32^, ^33^, ^34^, ^35^, ^36^ or the correction of *x*‐ray Computed Tomography (CT) based 2D proton radiographs.^37^, ^38^ Proton radiographs have also been proposed as a method of ensuring accurate patient positioning prior to treatment.^39^ Previous studies were carried out for the use case of measuring tumor position^40^, ^41^; however, these studies were based on simulated radiographs for a single event tracking detector design. These designs rely on the detection and reconstruction of individual protons^42^, ^43^ to limit the effects of multiple Coulomb scattering on image quality.^44^, ^45^ No single event ion imaging device has been implemented clinically, due to the complex detector setup, as well as the need to operate in particle rates^46^ $∼10^{6}$ Hz, well below clinical ion beams^47^, ^48^, ^49^ $∼10^{9}$ Hz.

An alternative method for ion radiography is to measure the integrated signal of each pencil beam and use this signal as a basis for image formation.^50^, ^51^, ^52^, ^53^ This approach suffers from worse image quality and generally higher imaging dose, (∼1cGy)^54^ than single event designs (∼10μGy).^48^ However, it is compatible with the particle rates of clinical ion beams, and is therefore a candidate of interest for visualizing tumor motion. Recently, physics‐based reconstruction models have been shown to improve image quality, though not to the level of single event systems.^55^, ^56^

This work shows the first instance of an integrated‐mode ion imaging detector being used to measure and monitor the position of a moving object, in a clinical beam line, with an anthropomorphic phantom, demonstrating its potential for use in particle therapy for lung cancer.

## 2 MATERIALS AND METHODS

**FIGURE 1:**
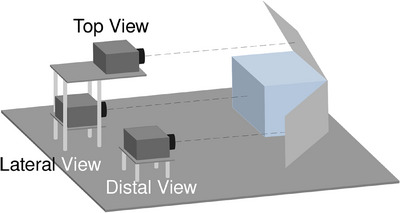
Schematic of the detector design, showing the monolithic scintillator being observed by three digital cameras. The top and distal cameras observe the scintillator through $45^{\circ }$ mirrors.

Experimental data was acquired at two ion beam therapy centers, using a monolithic plastic scintillator viewed from three sides (Distal, Lateral, and Top—see Figure 1) by digital cameras to capture the light from incident proton beams, similar to that used by Darne et al.^57^ This system was used to acquire radiographs of objects undergoing known motion. The images were reconstructed using a physics‐based back projection of the lateral and top views.^56^ The images were separated in frames and the centroid of the object was used to determine the potential accuracy of using proton radiographs for motion management. Additionally, the motion artifacts caused by the interplay between the scanning of the beam and the motion of the object were investigated by acquiring radiographs using different scanning patterns.

### 2.1 Detector design

The geometry of the proton radiography system used is shown in Figure 1. The detector's active component was a monolithic plastic scintillator (NUVIA Tech, Třebíč, Czech Republic) with dimensions of 25×25×20 ${\mathrm{cm}}^{3}$. The plastic scintillator was polystyrene based with an RSP of 1.043, as determined experimentally using a multilayer ionization chamber, Giraffe, (IBA Dosimetry, Schwarzenbruck, Germany). The scintillator was sanded and painted black, on three sides, to diffuse and absorb incident light respectively, and therefore reduce reflections from these faces. Three Charge‐coupled Device (CCD) cameras, ORYX‐10G‐51S5M (Teledyne FLIR, Wilsonville, OR, USA), were orientated to provide perpendicular views to cover the remaining three faces of the scintillator:

1. Distal view—covering the face of the scintillator opposite to the incident beam through a mirror behind the scintillator at a $45^{\circ }$ angle
2. Lateral View—covering the side face of the scintillator perpendicular to the incident beam
3. Top view—covering the top face of the scintillator, perpendicular to the incident beam through a mirror above the detector at a $45^{\circ }$ angle

Each camera was equipped with a LM6HC 6 mm focal length lens (Kowa, New York, NY, USA) focused in the center of the scintillator at a working distance of 42.5 cm. The optical field of view (FOV) was cropped to the edges of the scintillator, resulting in a final frame rate of 333 Hz and a pixel size of 0.4004 mm at the surface of the scintillator. All components were fixed in place to an optical breadboard and covered within an optical enclosure to minimize background light. Acquisitions were controlled through the Spinnaker SDK software (Teledyne FLIR, Wilsonville, OR, USA) and triggered using a Transistor‐Transistor‐Logic (TTL) input wire in the top camera. When this camera received a signal through the TTL input, the signal was propagated to the lateral and distal cameras. The initial input was generated using one of two methods depending on the ion beam therapy center where data was acquired:

1. A PDAPC2 amplified silicon photodiode (Thorlabs, Newton, NJ, USA) with a 1 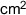 active area directed at the scintillator (Section 2.3).
2. The Trigger Next Spot signal from the accelerator (Section 2.4)

The input was processed through a microcontroller (Arduino, Somerville, MA, USA) to digitize the signal. In both cases, images of individual pencil beams were captured from each angle.

### 2.2 Experiment design

Two experimental setups were used for the work presented here. The first, an anthropomorphic thorax phantom was scanned in a clinical proton facility, with the aim of determining how accurately the position of a moving object can be determined. The second, a simplified lung model, was scanned in a clinical proton‐carbon facility, with the goal of investigating how the interplay between the beam scanning and object motion generates artifacts in the resulting images.

### 2.3 Position measurements

**FIGURE 2:**
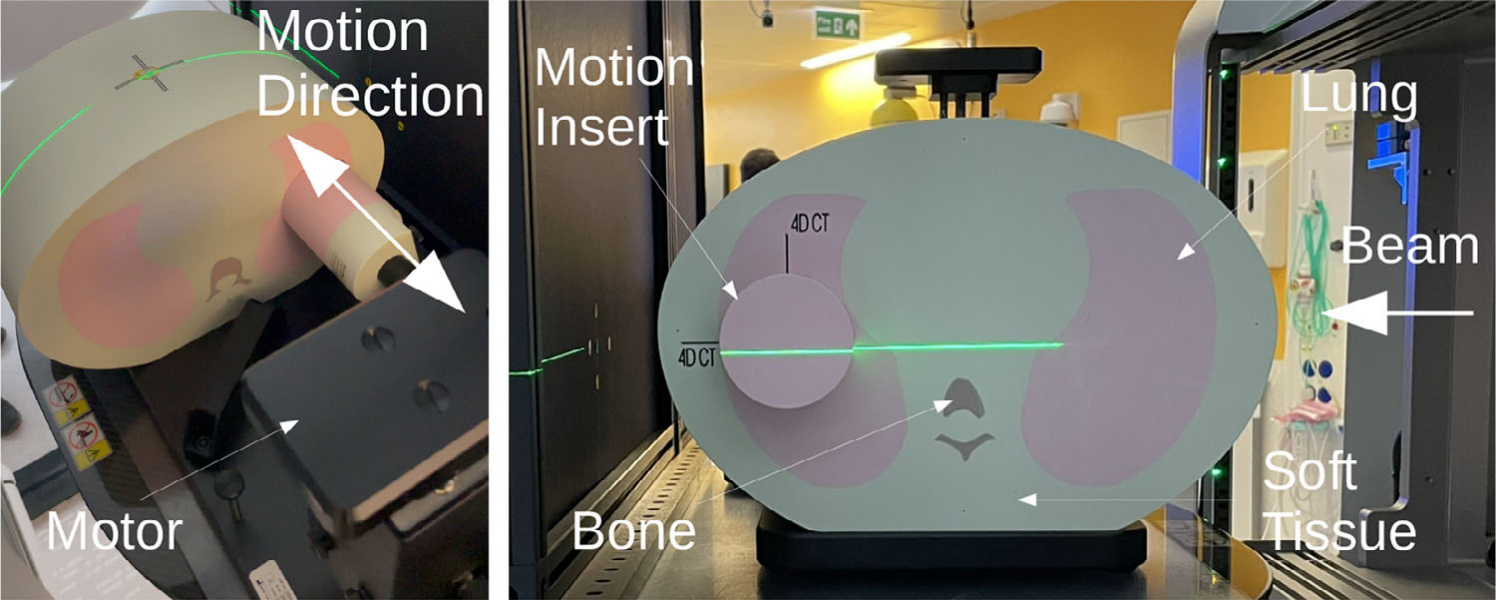
Experimental setup for the positional accuracy experiments using the anthropomorphic thorax phantom. The phantom is made up of materials mimicking Lung (0.21 g cm^−3^), Bone (1.20 g cm^−3^ Trabecular, 1.91 g cm^−3^ Cortical), and Soft Tissue (1.04 g cm^−3^). The motion insert was driven in a periodic motion by the motor.

The first phantom used was an anthropomorphic thorax phantom (CIRS Inc., Norfolk, VA, USA) with a spherical tumor insert which can be driven in a periodic motion using an electric motor (Figure 2). The detector was positioned so that the scintillator's surface aligned with the system isocenter. The phantom was placed between the detector and the nozzle, aligned laterally and vertically with the isocenter using the lasers which are accurate to within 1 mm.

The delivery system for these acquisitions was a clinical ProBeam (Varian Medical System, Palo Alto, California, USA) operated at an energy of 230 MeV giving a spot size of 7.8 mm full width at half maximum (FWHM) in air at the entrance of the scintillator. For this experiment, trigger signals from the accelerator were not available, and the photodiode system was used for acquisition triggering. To overcome the ProBeam's continuous scanning limitation that prevents resolving a single beam with a light‐trigger, we employed a workaround involving forced beam pause by skipping spot positions and performing multiple passes over the phantom, each time shifted by one spot position, at the cost of increased sampling time.

The minimum field size was set to encompass the tumor insert undergoing the largest amplitude of the programed motion (20 mm) with a 1 cm margin around the edge, with a fixed spot spacing of 5 mm. The beam delivery speed was not adjustable so to simulate faster acquisition speeds, the motion of the tumor insert was slowed. During the experiment, acquisitions were made with a variety of motion parameters and tumor insert sizes. The motion followed the waveform defined in Equation (1) over time, t, in the direction indicated in Figure 2.

(1) $$y=-2Acos^{4}\left(\frac{\pi t}{T}\right)+A$$

Where A is the amplitude of the defined motion, T is the period of the defined motion. Table 1 shows the insert size, field size of the acquisition, acquisition frame rate and motion parameters used in this experiment. For each measurement, 20 total frames were acquired.

**TABLE 1:** Results of the positional measurement accuracy, the MAE ±σ between the measured and expected position, for the different tumor insert sizes, and motion parameters.

Insert diameter (mm)	Amplitude (mm)	Period (s)	Field size (cm^2^)	Frame acquisition time (s)	MAE (mm)
30	20	6	9 × 5	1.4	3.1 ± 4.5
30	20	12	9 × 5	1.4	2.2 ± 2.9
30	20	24	9 × 5	1.4	1.0 ± 1.4
30	15	24	9 × 5	1.4	0.9 ± 1.3
30	10	24	9 × 5	1.4	0.7 ± 0.8
20	20	24	8 × 4	1.0	1.8 ± 0.9
10	20	24	7 × 3	0.7	1.9 ± 2.3

Abbreviation: MAE, mean absolute error.

Each series of pencil beams was first sorted and reconstructed (see Section 2.5) so that they form individual radiograph frames. Then, an intensity‐based threshold was automatically determined using Otsu's method^58^ to find the position of the insert in each frame. To mitigate the impact of background variation on the threshold, the image was divided into 11 horizontal strips and a threshold value determined for each strip individually. The shape of the insert identified from each strip was combined to obtain the full shape. The centroid of the identified shape was taken as the sphere's position and an offset from the center of the FOV was determined in millimeter. This position was then compared to the expected position of the sphere, calculated using Equation (1), at the time of each frame, taken to be the time of acquisition of the median image in each frame.

### 2.4 Motion artifacts

**FIGURE 3:**
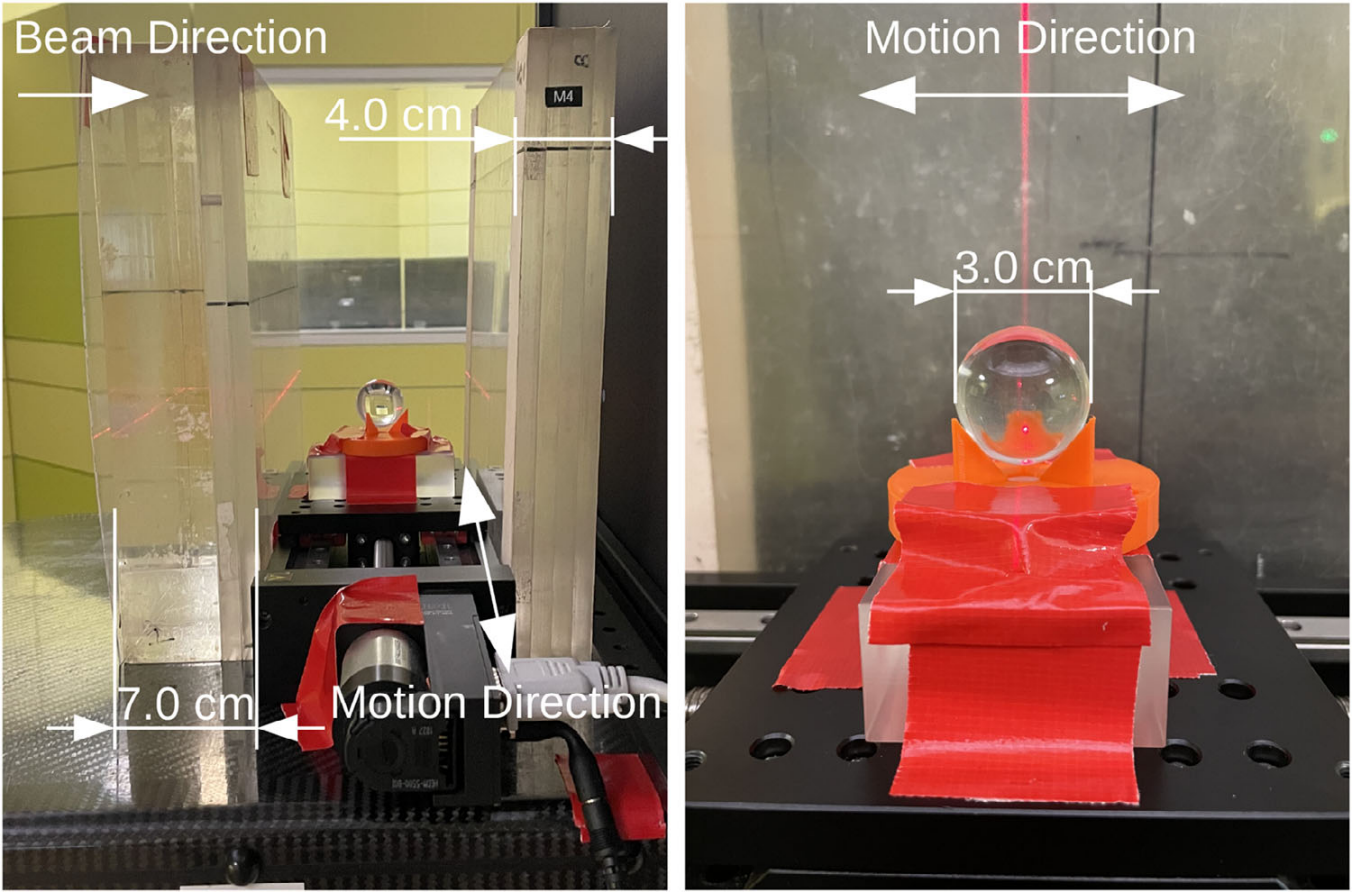
Experimental setup for the motion artifact experiments. The PMMA sphere is mounted on in a holder atop a controlled motion table. 7 cm of PMMA is placed in front of the motion table and 4 cm of PMMA behind to create a simplistic lung model.

The second set of experiments was performed at the Marburger Ionenstrahltherapie‐Zentrum (MIT) Siemens synchrotron with a proton beam of 180 MeV and 9.3 mm FWHM in air at the isocenter. The setup for this experiment was a simplified model of a lung tumor in motion using slabs of polymethyl methacrylate (PMMA) (7.0 cm proximal, 4.0 cm distal) and a water equivalent sphere (⌀ = 30 mm) placed between them on a motorized table (M‐404 PD linear stage, Physik Instrumente, Karlsruhe, Germany), as shown in Figure 3. The 30 mm PMMA sphere was placed on a programable lateral motion platform within a 3D‐printed holder. During acquisition, the table underwent a periodic motion with amplitude of 20 mm and period of 4 s following Equation (1). Only acquisitions within the first spill were analyzed. The accelerator signals were available for this system, so the next spot signal was used for acquisition triggering.

The PMMA sphere center was aligned with the center of the scintillator, which was placed with its front face aligned with the origin of the delivery system using the lasers which are accurate to within 1 mm. Sequential 6× 6 ${\mathrm{cm}}^{2}$ fields were delivered using vertical, horizontal, and spiral scanning patterns (Figure 8). Unlike conventional *x*‐ray radiography, a pencil‐beam ion radiograph is not acquired instantaneously, but is subject to the beam scanning speed. Since the underlying object was in motion throughout the acquisition, the limited temporal resolution caused motion artifacts. To investigate the effects of these artifacts, the following beam scanning approaches were explored, horizontal scanning, vertical scanning, and spiral scanning.

**FIGURE 4:**
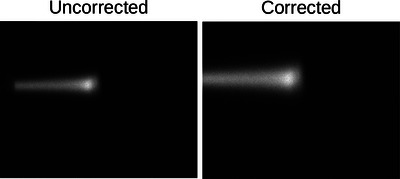
Demonstration of the effect of the optical corrections on the raw pencil beam images from the acquisition. This figure shows the same pencil beam re‐projected onto the front face of the scintillator through the perspective correction.

**FIGURE 5:**
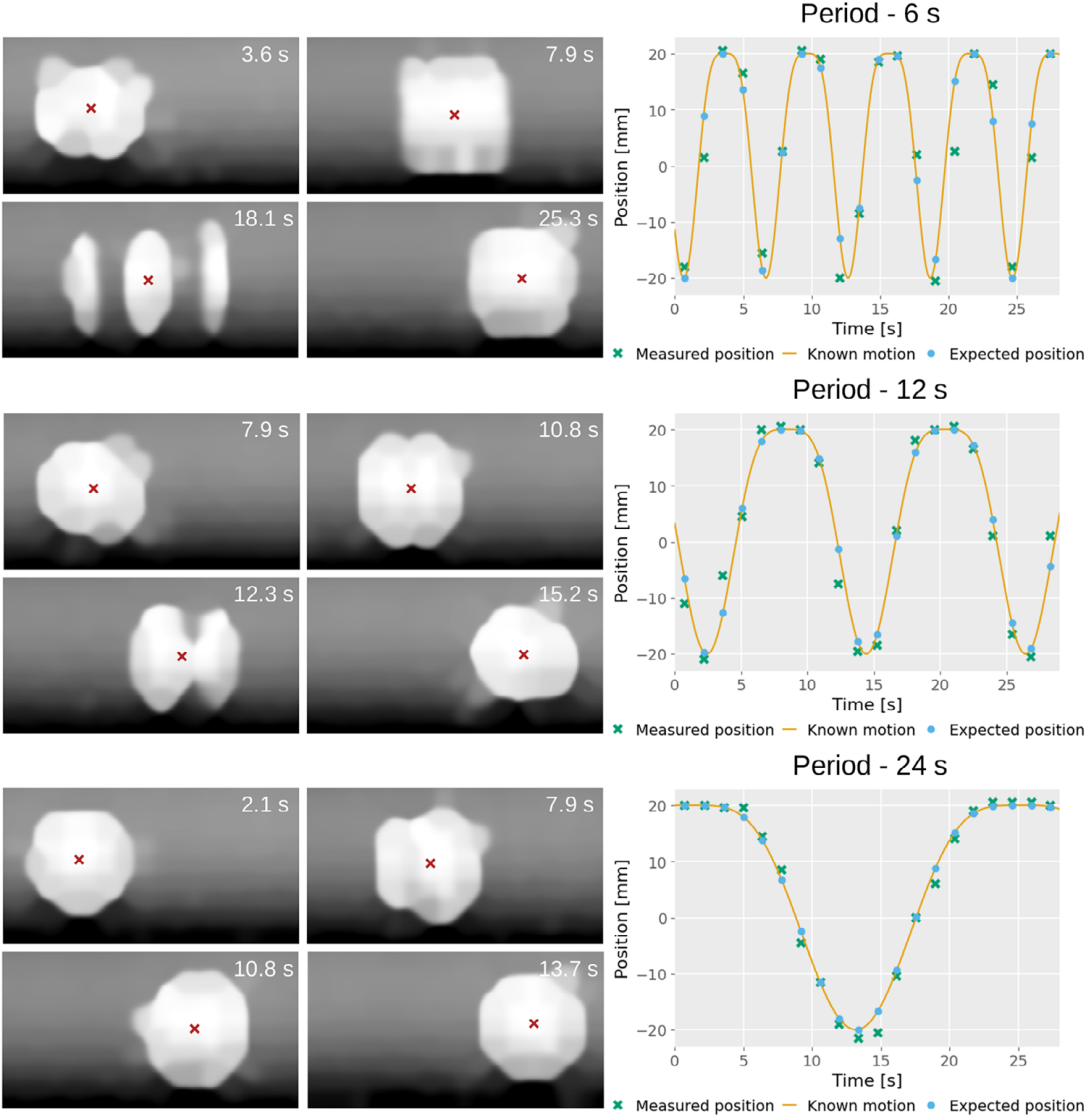
Plots of the measured and expected position of the tumor insert over time for the 30 mm tumor insert, with a 20 mm amplitude for different motion periods. On the left, are examples of frames for each plot, with the measured center of the insert indicated by the red cross. The time of each acquisition is displayed in the top corner of the image.

**FIGURE 6:**
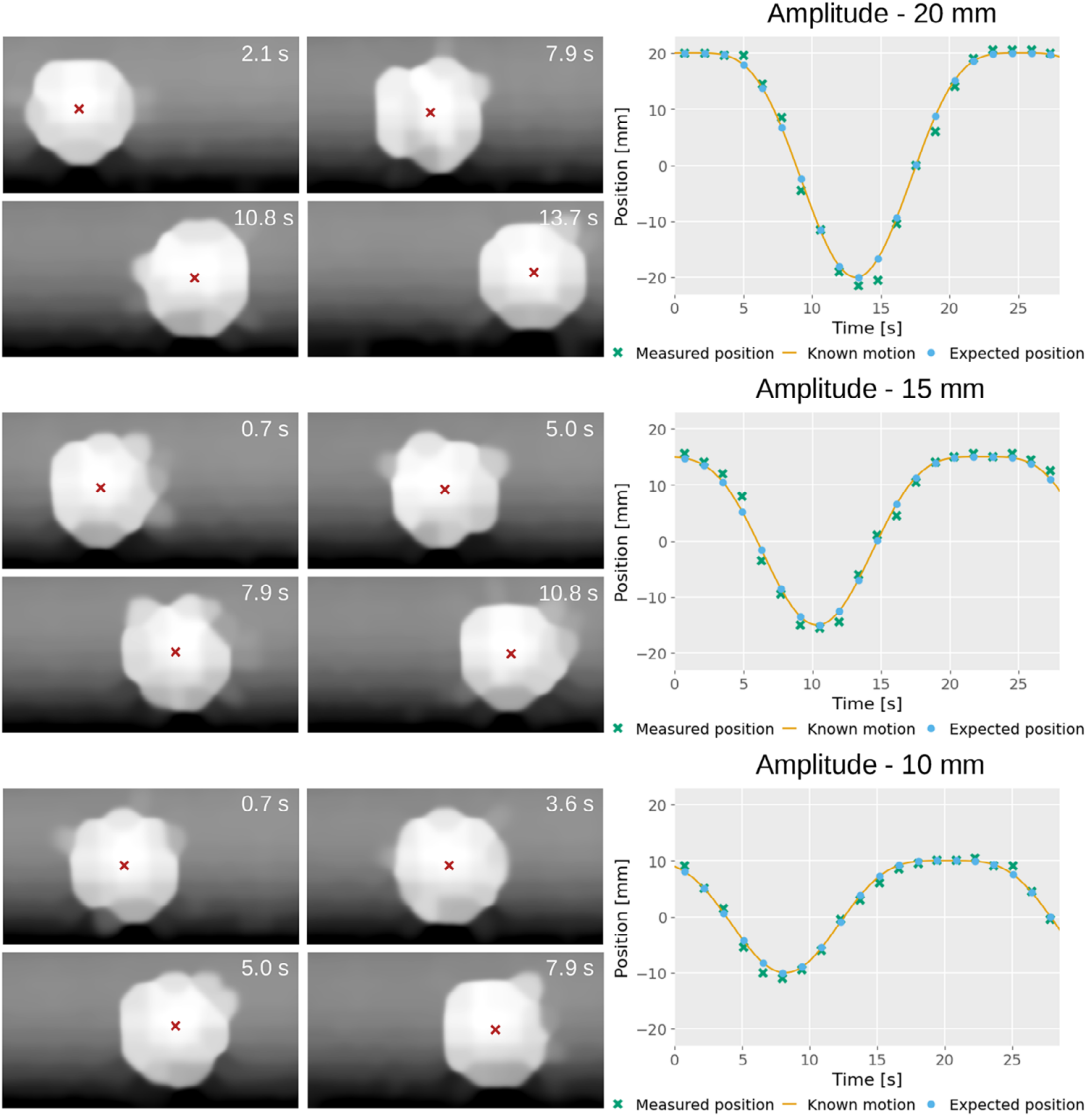
Plots of the measured and expected position of the tumor insert over time for the 30 mm insert with a 24 s motion period for different amplitudes. On the left, are examples of frames for each plot, with the measured center of the insert indicated by the red cross. The time of each acquisition is displayed in the top corner of the image.

**FIGURE 7:**
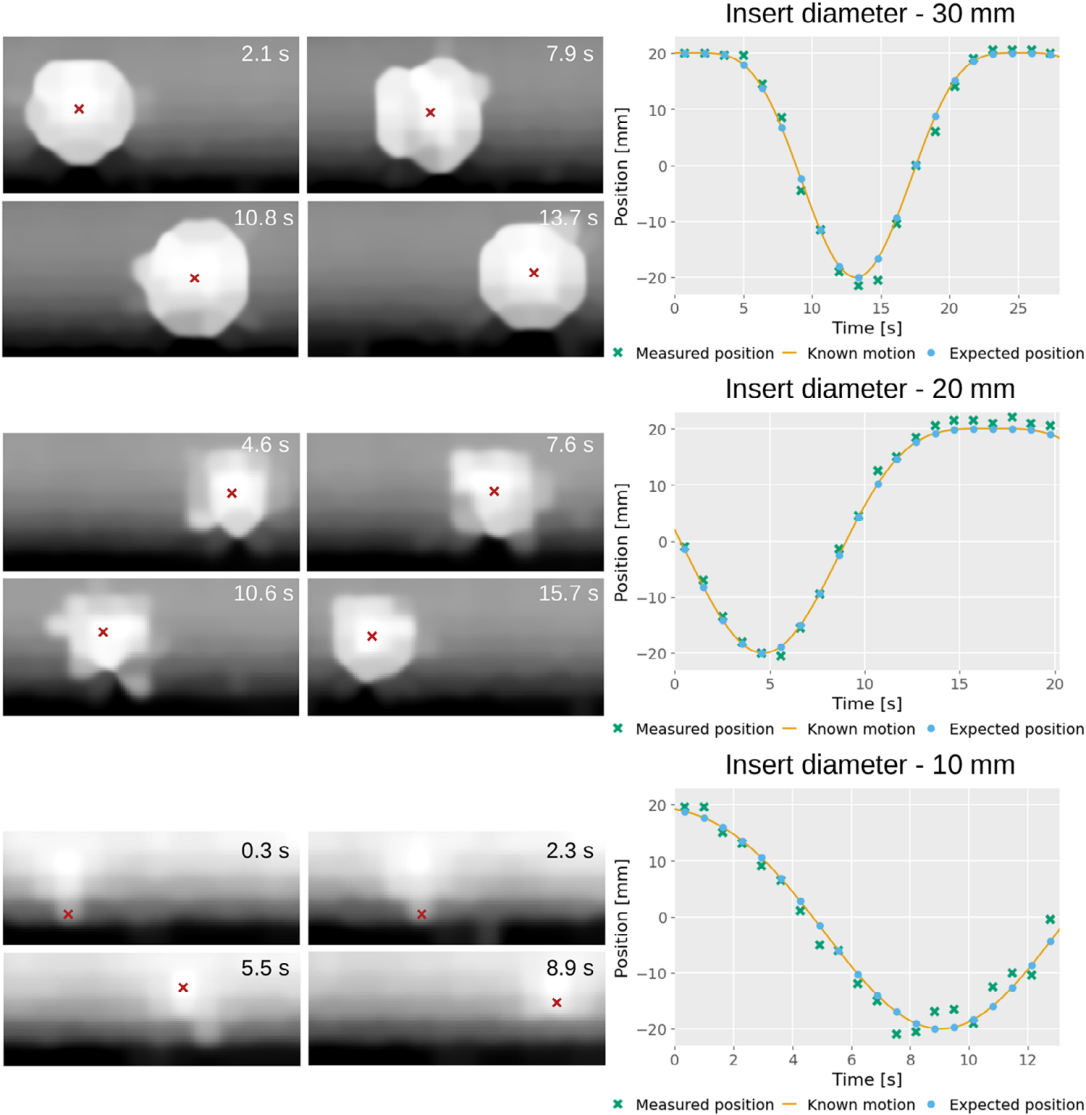
Plots of the measured and expected position of the tumor insert over time for the 20 mm amplitude and 24 s motion periods with different tumor insert sizes. On the left, examples of frames for each plot, with the measured center of the insert indicated by the red cross. The time of each acquisition is displayed in the top corner of the image.

**FIGURE 8:**
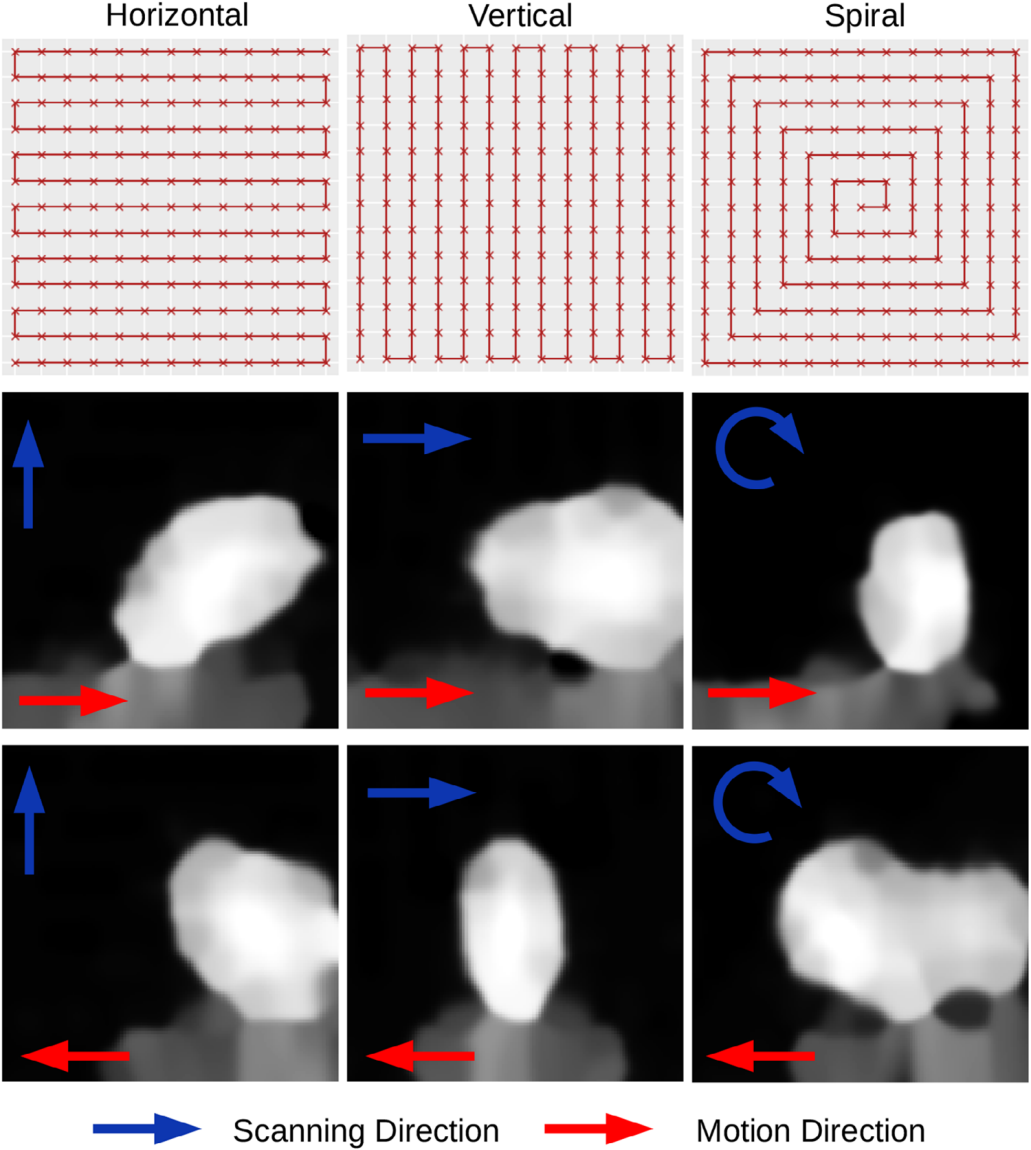
Demonstration of the effect of different scanning patterns on the resulting image when imaging a moving sphere with proton radiography. The first row shows the scanning pattern, The beam started at one end of the red line and scanned following the red line with the ‘x’ set spot positions for a frame. The direction was then reversed for the next frame. The first column shows the effect of imaging in parallel with the direction of motion, the middle shows the effect of sampling perpendicular to the object motion and the third column shows the effect of imaging in a spiral pattern. The direction that the beam moved through the pattern and the direction of motion are indicated by the arrows.

### 2.5 Reconstruction

The acquired camera images from the top and lateral views were first corrected for optical effects. Distortion effects, resulting from the lens, are corrected for using camera calibration techniques described in Robertson et al.^59^ The procedure was carried out for each camera individually using the acquisition settings as intended for imaging to determine the calibration matrix for each camera‐lens combination. Optical vignetting was corrected using a cos^4^ attenuation approximation described by Ray^60^ Geometry and refraction corrections were applied to reproject all pencil beams as if they entered the scintillator at the face nearest to each respective camera (Figure 4).

#### 2.5.1 Physics‐based back projection

The proton radiograph reconstruction followed the 2D Lateral method proposed in Simard et al.^56^ Shortly, for each pencil beam, a series of individual pristine peaks were extracted from the top and lateral view. These peaks were then converted into the WET of the material the pristine beam has traversed. The lateral and vertical position information of the original pencil beam, as extracted from the top and lateral cameras, was then used to reproject the WET values while accounting for the multiple scattering.

## 3 RESULTS

Table 1 shows the MAE between the measured position of the insert and the expected position, according to the known motion, at the time of the frame for the experiments in Section 2.3. Plots comparing the measured position and expected position, over time, are presented in Figures 5, 6, 7 for each of the investigated variables: period, amplitude and tumor size. Examples of each are shown alongside the plots along with their time of acquisition, chosen to show the insert in a variety of positions. The motion artifacts from each of the scanning patterns from Section 2.4 are shown in Figure 8.

### 3.1 Position measurements

As expected, the accuracy of measured positions improves with slower tumor motion, achieving 3.1, 2.2, and 1.0 mm for 6, 12, and 24 s periods, respectively, using the same tumor size and amplitude (30 and 20 mm respectively). Accuracy also improves with smaller motion amplitudes (1.0, 0.9, and 0.7 mm for 20, 15, and 10 mm, respectively) due to slower tumor motion at a constant period (24 s). Similarly, positional measurements for different tumor sizes achieve good accuracy (1.0, 1.8, and 1.9 mm for 30, 20, and 10 mm respectively), although smaller tumors exhibit worse accuracy.

### 3.2 Motion artifacts

Figure 8 illustrates the impact of different scanning patterns on motion artifacts. Each pattern has two distinct effects depending on the direction of motion of the sphere relative to the direction the beam moves through the pattern. The horizontal scanning pattern skews the sphere diagonally, in the direction that the object is moving. The vertical scanning pattern contracts the object if the motion is in the opposite direction to the scan direction, or expands the sphere if they are in the same direction. The spiral pattern exhibits more extreme effects, with the sphere being sampled in two places within a single frame due to it being measured first on the edge of the frame and then again when it moves into the center. Alternatively, it is only partially sampled in the situation where it is first sampled towards the edge of the frame but moves out of the center while the beam is sampling there. When the machine trigger is not available for image acquisition synchronization, the larger distance between spots necessary for optically‐triggered acquisition results in distortion of the sphere's appearance into separate sections. Approximating the appearance of the sphere in the image generated by each scanning pattern as an ellipse, we can calculate the eccentricity and quantify how much it has deviated from a circle, which would yield an eccentricity value of 0. The maximum and minimum eccentricity respectively for the horizontal pattern were 0.8 and 0.7, for the vertical pattern they were 0.9 and 0.6 and for the spiral pattern they were 0.9 and 0.7.

## 4 DISCUSSION

This work marks the first experimental demonstration of tumor tracking accuracy with continuous proton imaging. Quantitative analyses with an anthropomorphic lung phantom were used to assess the possibilities and limitations of this approach. Measurements with a moving tumor insert on a programable linear stage were used to investigate imaging defects due to the interplay of pencil beam scanning with the object motion.

When evaluating the performance of the tracking system, it is important to know the required accuracy for use in treatment delivery. Steinsberger et al.^25^ proposed a 4D particle therapy planning method for mitigating tumor motion with 4D plan optimization and residual beam tracking, concluding that a positional accuracy of 1.9 mm or better would be required for high dosimetric fidelity of the delivery when a tumor is undergoing irregular motion and carbon ions are used for treatment. They also note that for proton beams, the increased scattering results in larger spot sizes and reduced sensitivity to motion. The results of the work, presented here, demonstrates that integrated‐mode proton radiography can achieve this accuracy in principle. Due to current technical limitations at the Varian ProBeam center, such as the lack of a next point trigger signal, more realistic breathing motions (6 s period) do not achieve this positional accuracy. However, tracking accuracy of 1 mm was achieved for the 30 mm tumor insert when using slower breathing periods, imitating a factor four faster acquisition speed. It is not known if 3.1 mm would be sufficient accuracy to apply to proton beam treatments, but is likely close based on the requirements of carbon ions.

Studies on the effect of tumor motion on the delivered dose distribution have determined that the amplitude of the motion has the biggest impact.^61^, ^62^, ^63^ The amplitudes in this study are much larger and therefore would have a greater clinical effect; however, such large breathing motion is not typical.^64^ For smaller motion amplitudes, the technique in this work has shown improved accuracy, indicating that it would do better on more clinically relevant breathing motions. Although it has been previously indicated tumor motions below 10 mm do not significantly alter the delivered dose,^61^, ^63^ dose homogeneity is still reduced^62^ which tumor tracking should help mitigate.

There was a reduction in the positional accuracy with smaller tumor inserts. This reduction can be attributed to undersampling artifacts from fewer pencil beams crossing the insert, which reduces the ability for centroid to be accurately determined. This is most noticeable in the poor quality of the 10 mm tumor insert images for which, at 5 mm beam spacing, only a maximum of four pencil beams will cross the projection of the tumor. Though simplified, the phantom used in this study shows that tumors of 10 mm could be detected in this method in a realistic scenario. The limit in this case would be the ability to visualize the tumor against the background. This reconstruction method has been shown to resolve contrasts of 0.5% for objects of 1 cm.^56^ More advanced methods of defining the tumor center, such as the Retina U‐net,^65^ would likely return better accuracy and improve its applicability in the clinic.

The impact of motion artifacts in the resulting images has been demonstrated. Non‐continuous scanning patterns created the worst artifacts, breaking up the sphere's image in some cases. Horizontal and vertical scanning had the least effect on the sphere's appearance, but still distorted its overall shape, necessitating consideration of the target area when used for plan adaptation. The horizontal scanning pattern had the most consistent range of eccentricity, indicating that the distortions it provides are consistent over the whole acquisition. The spiral pattern was worse overall than the other patterns, as its eccentricity was consistent and higher than the others. The vertical scanning pattern was the most inconsistent, with the largest range, but also had the lowest eccentricity value (0.6). An optimal solution would involve using a pattern that samples the tumor's motion area as closely and quickly as possible, potentially devising a custom pattern online after an initial scout pattern. Further work in this area is needed to optimize acquisition parameters to the specific task of motion monitoring. Tanaka et al.^66^ proposed a gated image acquisition to reduce motion artifacts during the long acquisitions of single event proton radiography. However, this method would not be applicable for standalone tumor tracking due to the need for an additional independent tracking system.

In general, faster acquisition leads to fewer motion artifacts and improved accuracy of the position measurement. This suggests that a passively scattered proton beam may yield the best results for proton radiography based tumor tracking, as it would remove the interplay between the beam scanning and the tumor motion. However, this would likely come with a reduction in image quality, which may hinder the tracking accuracy, due to the limitations imposed by multiple Coulomb scattering as the passive beam delivery would prevent the use of the advanced reconstruction techniques employed in this work. It is expected that the heterogenous anatomy of the lung would be hardly visible, providing little information for tracking.

A simplified thorax anatomy with a regular breathing motion was used for this investigation. In reality, the anatomy would be more heterogenous, with ribs and lung substructures. Additionally, we know that patient breathing motions can change over time and have discontinuities.^67^, ^68^ Studies have shown through simulation that good image quality can be achieved in this anatomy, though a larger and still regular tumor shape was used.^56^ The achievable image quality in a clinical scenario with the minimum achievable imaging dose needs to be investigated to determine its clinical applicability. Additionally, the appearance of irregular tumor shapes, which will have a less defined edge, undergoing motions including discontinuities should be studied to see how they affect motion artifacts and the accuracy to which the tumor position can be determined. Finally, to use the system to dynamically treat lung tumors, latencies need to be assessed including image reconstruction times and the time taken to switch to the treatment energy would need to be taken into account either through prediction of the motion in between frames^69^ or additional target margins.

In this study, the achievable scanning speed was limited by the need to trigger acquisitions using a photodiode. This limitation could be overcome, if a next point trigger signal was available at the facility. Based on a raster scanning delivery method of the Varian ProBeam, with access to trigger signals, the time to scan a single frame of the 9 × 5 cm^2^ field used for the 30 mm tumor would be 0.5 s. At this speed, the accuracy achievable would be in the range of the 12 s acquisition (2.2 mm) and future increases in scanning speed, through a reduced minimum spot time or faster scanning magnets, would improve this accuracy. It can therefore be concluded that dynamic proton radiography would indeed be a feasible tool to guide motion mitigated treatment delivery for particle therapy in terms of accuracy.

## 5 CONCLUSIONS

This study has shown that it is possible to accurately measure the position of moving targets using integrated‐mode proton radiography. With a breathing motion period of 6 s, a positional accuracy of 3.1 mm was achieved, which may be accurate enough for 4D planning techniques used in particle therapy. When the breathing period was increased to 24 s this improved to within 2 mm. This accuracy improvement is the result of reduced motion artifacts. This has the implication that with acquisition speeds resulting in 2–4 frames per second, faster than was possible for these experiments, this accuracy could be achieved for a realistic breathing motion. The pattern of beam scan for the acquisition also has an impact on the appearance of motion artifacts, and more work is required to find the optimal pattern for a given tumor motion. Regardless, acquisition speed seems to be the determining factor in the achievable accuracy of using integrated‐mode ion imaging for tumor tracking in proton therapy.

## CONFLICT OF INTEREST STATEMENT

The authors declare no conflicts of interest.
